# Osseous free flap vs. Bridging plate mandibular reconstruction: a retrospective cohort study on perioperative complications of 335 patients

**DOI:** 10.1007/s00784-026-06753-7

**Published:** 2026-01-17

**Authors:** Philipp Lampert, Jakob Fenske, Kilian Kreutzer, Henri Kreiker, Norbert Neckel, Steffen Koerdt, Max Heiland, Carsten Rendenbach

**Affiliations:** Corporate Member of Freie, Department of Oral and Maxillofacial Surgery, Charité – Universitätsmedizin Berlin, Universität Berlin and Humboldt-Universität zu Berlin, Berlin, Germany

**Keywords:** Microvascular reconstruction, Mandibulectomy, Patient-specific reconstruction, Customized mandibular implant, Dental rehabilitation, 3D-printed osteosynthesis

## Abstract

**Objectives:**

This study compared postoperative complication rates between bridging plate reconstruction with soft tissue free flaps and osseous free flaps for segmental mandibular defects.

**Materials and methods:**

This retrospective study compared postoperative outcomes between both techniques in 335 reconstructions of segmental mandibular defects operated over 8 years. The N-1 χ^2^-test, Fisher’s exact test and Mann-Whitney-U test were used to test for statistical significance after mode and mean imputation were performed on independent variables with missing data.

**Results:**

Patients who received bridging plate reconstructions were significantly older (74.4 ± 10.5 vs. 63.4 ± 10.6 years, *p* < 0.001) and had higher rates of hypertension (67.6% vs. 40.6%, *p* = 0.002), atherosclerosis (27.0% vs. 12.8%, *p* = 0.020), and prophylactic anticoagulation (21.6% vs. 8.7%, *p* = 0.014). Treatment indications differed significantly, with malignant tumors in 59.5% vs. 78.5% and osteonecrosis in 40.5% vs. 21.5% of bridging plate and osseous reconstructions, respectively (*p* < 0.001). Mortality (24.3% vs. 10.1%, *p* = 0.011) and pneumonia rates (13.5% vs. 4.7%, *p* = 0.029) were higher in the bridging plate group. Plate exposure (21.6% vs. 24.8%, *p* = 0.669), fixation failure (5.4% vs. 6.0%, *p* = 0.878), and early flap loss (5.4% vs. 4.4%, *p* = 0.773) showed no significant differences. Bridging plate reconstructions had shorter surgery times (8.4 ± 2.0 vs. 9.6 ± 2.2 h, *p* = 0.002) and fewer wound infections (13.5% vs. 30.9%, *p* = 0.028).

**Conclusions:**

Our findings emphasize the value of bridging plate reconstruction as a pragmatic and effective option when osseous reconstruction is contraindicated and advocate for personalized treatment strategies.

**Clinical relevance:**

These findings validate bridging plate reconstruction as a safe and pragmatic alternative for patients with contraindications to osseous flaps, such as advanced age or significant comorbidities. The comparable complication rates and shorter operative times support its use in personalized treatment planning.

**Supplementary Information:**

The online version contains supplementary material available at 10.1007/s00784-026-06753-7.

## Background

Reconstruction of segmental mandibular defects is essential for restoring esthetics, oral function and preventing complications like glossoptosis, malocclusion and dysphagia. Free vascularized bone grafts are widely considered the gold standard for these reconstructions, as they enable removal of osteosynthesis hardware following bone healing, thereby allowing for full prosthetic and dental rehabilitation through placement of dental implants in the transplanted bone [1, 2].

However, osseous flap reconstruction is not always feasible or appropriate. Harvesting vascularized bone grafts is inherently more invasive and time-consuming than harvesting soft tissue flaps like the radial forearm (RFF) or anterolateral thigh flap (ALT), making this approach less suitable for patients with limited life expectancy due to disease progression, significant comorbidities, or advanced age [3]. In addition, the increased donor site morbidity often associated with osseous transplants may preclude this option for patients who wish to maintain certain physical activities. In cases requiring adjuvant radiotherapy, the transplanted bone is at additional risk of osteoradionecrosis [4]. Furthermore, adequate vascular supply to the donor site is essential but may be compromised in patients with atherosclerosis, thrombosis, or other vascular pathologies [5].

**Fig. 1:**
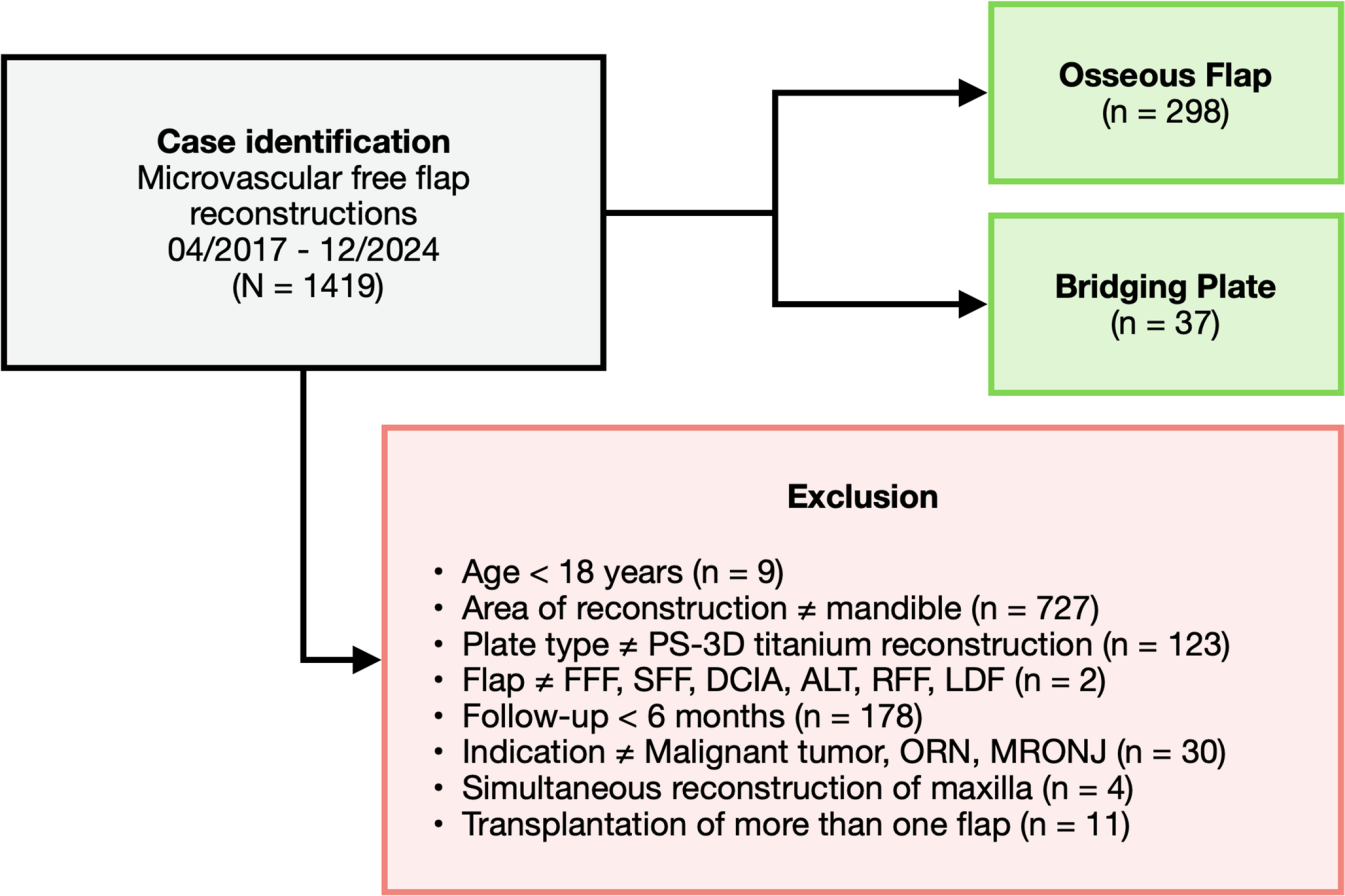
Visualization of the patient inclusion process. *FFF = fibula free flap*,* SFF = scapula free flap*,* DCIA = deep circumflex iliac artery flap*,* ALT = anterolateral thigh flap*,* RFF = radial forearm flap*,* LDF = latissimus dorsi flap*

When osseous reconstruction is contraindicated, soft tissue flaps can be used to cover a 3.0 mm load-bearing bridging reconstruction plate to maintain mandibular continuity (Fig. 1). However, the primary limitation of this technique is the complication of dental rehabilitation, as the absence of bone precludes conventional dental implant placement in the defect area. Recent innovations in reconstruction plate design have shown promise in pilot studies by integrating screw holes for dental implants, but have not yet gained widespread clinical adoption [6, 7]. In addition, bridging plate reconstructions have been shown to result in significantly higher rates of plate exposure than osseous reconstructions in a recent meta-analysis (20% vs. 10%, *p* < 0.01), although heterogeneity between individual studies remained high [8].

Despite bridging plate reconstructions being an established alternative to osseous free flaps, comparative studies evaluating postoperative outcomes between these approaches remain limited. We conducted a retrospective cohort study comparing postoperative complication rates between patient-specific bridging plate reconstructions with soft tissue free flaps and osseous free flap reconstructions in a large, single-center patient cohort.

## Methods

### Study design

This retrospective single-center cohort study was conducted at the Department of Oral and Maxillofacial Surgery at Charité – Universitätsmedizin Berlin. Patients who received a microvascular free flap reconstruction between April 2017 and December 2024 were screened for study eligibility. Follow-up data was analyzed until February 2025. Ethical approval was obtained from the responsible ethics committee at Charité – Universitätsmedizin Berlin (EA2/138/18).

### Inclusion and exclusion criteria

Inclusion criteria were microvascular reconstructions of segmental mandibular defects using a patient-specific 3D-printed (PS-3D) titanium reconstruction plate. Flap donor sites other than fibula (FFF), scapula (SFF), deep circumflex iliac artery (DCIA), radial forearm (RFF), anterolateral thigh (ALT) and latissimus dorsi (LDF) were excluded due to low occurrences. Patients who received more than one flap in the same surgery were excluded. Likewise, patients with osteosynthesis other than PS-3D titanium reconstruction plates were not included to ensure a homogenous study population. Patients under the age of 18 and cases with missing data among predictor variables were also excluded. Cases with missing data in the outcome variable were excluded only in the respective analysis. The minimum follow-up time was 6 months.

### Procedures

The PS-3D titanium reconstruction plates were designed following a previously described workflow [9]. For osseous reconstructions, PS-3D reconstruction plates with a thickness of 2.0 mm were fixed with bicortical 2.0 mm non-locking screws. For bridging plate reconstructions, PS-3D reconstruction plates with a thickness of 3.0 mm were fixed with bicortical 2.7 mm locking screws. All plates were manufactured by KLS Martin SE & Co. KG (Tuttlingen, Germany). Each patient received prophylactic anticoagulation, beginning during surgery and continuing throughout the postoperative hospital stay. Additionally, prophylactic intravenous antibiotics (ampicillin/sulbactam) were administered for one week postoperatively.

### Data acquisition

REDCap electronic data capture tools, hosted at Charité – Universitätsmedizin Berlin, were used for data collection [10]. Medical charts, the surgeon’s report and outpatient follow-up documentation were retrospectively reviewed for patient-, treatment- and complication-related information.

### Statistical analysis

Data engineering and statistical analysis were performed using the Python Programming Language (version 3.11). The SciPy Stats module and Scikit-learn package were used for statistical functions [11, 12]. Mode and mean imputation were used to impute missing data among independent variables. The highest percentage of missing data was found for the treatment indication variable at 4.3%.

Patients who received a FFF, SFF or DCIA free flap were allocated to the *osseous* group. Patients who received an ALT, LDF or RFF covering a 3.0 mm bridging plate were assigned to the *bridging plate* group.

The N-1 χ^2^-test, Fisher’s exact test and Mann-Whitney-U test were used to test for statistical significance [13]. Effect sizes are shown using the *φ-coefficient* for boolean variables, *pearson correlation coefficient* for numeric variables and *Cramer’s V* for categorical variables with more than two levels. Kaplan-Meier survival analysis was performed for overall survival, with between-group comparison using the log-rank test. A stratified analysis restricted to patients with malignant tumors was conducted to address potential confounding by indication. The study’s level of significance was set at *p* ≤ 0.05.

### Outcome definitions

All outcomes were assessed throughout the entire follow-up period. Flap loss was further divided into *early* and *late* flap loss based on a 6-week threshold to account for the differing etiology. While early flap losses are often caused by vascular complications, late flap losses are rare and more likely due to external factors like adjuvant radiotherapy [14]. Wound infection was defined as infection at the recipient site requiring antibiotic treatment beyond routine prophylaxis or surgical intervention. Plate exposure was defined as visible titanium hardware through oral mucosa or cervical skin at any point during follow-up. Fixation failure included radiographically confirmed plate fracture or screw loosening. Osseous non-union was not included as an outcome variable, since bridging plate reconstructions are not at risk of this complication.

Grouped outcome variables were created to combine related complications:

- *Soft tissue complications* were defined as any of the following occurring at the flap recipient site: wound healing disorder (WHD), partial necrosis, wound infection, salivary fistula, bone exposure, plate exposure.
- *Donor site complications* were defined as any of the following occurring at the flap donor site: WHD, partial necrosis, wound infection, seroma.
- *Fixation failure* was defined as plate fracture and/or screw loosening.

## Results

### Patient inclusion

1419 microvascular free flap reconstructions were performed during the 8-year study period and screened for eligibility. Cases were excluded as per the previously described inclusion criteria (Fig. 1). The final study collective comprised 335 cases.

The characteristics of the study population are shown in Table 1. Patients receiving a bridging plate reconstruction were significantly older (74.4 ± 10.5 vs. 63.4 ± 10.6 years, *p* < 0.001) and had significantly higher rates of hypertension (67.6% vs. 40.6%, *p* = 0.002) and atherosclerosis (27.0% vs. 12.8%, *p* = 0.020). Additionally, they were significantly more often receiving prophylactic anticoagulation (21.6% vs. 8.7%, *p* = 0.014) and antiplatelet therapy (32.4% vs. 12.8%, *p* = 0.002). Patients in the *bridging plate* group had also significantly more often undergone prior reconstructive surgery with soft tissue flaps (24.3% vs. 11.7%, *p* = 0.033). However, there was no significant difference between groups regarding prior reconstructions with bone grafts. While malignant tumors represented the most common indication in both groups, they were significantly less prevalent among patients receiving bridging plate reconstructions, with osteoradionecrosis and medication-related osteonecrosis of the jaw (MRONJ) being more frequent indications in this group (*p* < 0.001).

**Table 1:** Patient characteristics

		Bridging Plate	Osseous Flap	*p*-value	Effect Size	Overall
		*n* = 37 (11.0%)	*n* = 298 (89.0%)			*N* = 335
Female vs. Male		11 (29.7%)	116 (38.9%)	0.278	φ = 0.059	128 (38.0%)
Age (years)		74.4 ± 10.5	63.4 ± 10.6	< 0.001	*r* = 0.304	64.6 ± 11.1
BMI (kg/m^2^)		23.9 ± 3.7	23.9 ± 4.5	0.938	*r* = 0.004	23.9 ± 4.4
Nicotine abuse		15 (40.5%)	122 (40.9%)	0.963	φ = 0.003	137 (40.9%)
Alcohol abuse		9 (24.3%)	64 (21.5%)	0.693	φ = 0.022	73 (21.8%)
Hypertension		25 (67.6%)	121 (40.6%)	0.002	φ = 0.170	146 (43.6%)
Atherosclerosis		10 (27.0%)	38 (12.8%)	0.020	φ = 0.128	48 (14.3%)
Prior Flap						
	Non-Bony	9 (24.3%)	35 (11.7%)	0.033	φ = 0.117	44 (13.1%)
	Bony	7 (18.9%)	37 (12.4%)	0.270	φ = 0.060	44 (13.1%)
Prior anticoagulation		8 (21.6%)	26 (8.7%)	0.014	φ = 0.134	34 (10.1%)
Prior antiplatelet drugs		12 (32.4%)	38 (12.8%)	0.002	φ = 0.173	50 (14.9%)
Indication				< 0.001	CV = 0.219	
	Malignant tumor	22 (59.5%)	234 (78.5%)			256 (76.4%)
	ORN	8 (21.6%)	53 (17.8%)			62 (18.4%)
	MRONJ	7 (18.9%)	11 (3.7%)			18 (5.4%)

*φ = phi coefficient*,* r = pearson correlation coefficient*,* CV = Cramer’s V*,* ORN = osteoradionecrosis*,* BMI = body mass index*,* MRONJ = medication-related osteonecrosis of the jaw*

Treatment-related characteristics are shown in Table 2. Surgery duration was significantly shorter for bridging plate reconstructions (8.4 ± 2.0 vs. 9.6 ± 2.2 h, *p* = 0.002). Preoperative radiotherapy was received more frequently in the *bridging plate* group (43.2% vs. 28.2%, *p* = 0.059), while adjuvant radiotherapy was significantly more common in the *osseous* group (41.3% vs. 24.3%, *p* = 0.047).

**Table 2:** Treatment characteristics

		Bridging Plate	Osseous Flap	*p*-value	Effect Size	Overall
		*n* = 37 (11.0%)	*n* = 298 (89.0%)			*N* = 335
Radiotherapy						
	None	14 (37.8%)	98 (32.9%)	0.548	φ = 0.033	112 (33.4%)
	Anamnestic	16 (43.2%)	84 (28.2%)	0.059	φ = 0.103	100 (29.9%)
	Adjuvant	9 (24.3%)	123 (41.3%)	0.047	φ = 0.109	132 (39.4%)
Chemotherapy						
	None	21 (56.8%)	185 (62.1%)	0.531	φ = 0.034	206 (61.5%)
	Anamnestic	12 (32.4%)	59 (19.8%)	0.077	φ = 0.097	71 (21.2%)
	Adjuvant	6 (16.2%)	62 (20.8%)	0.513	φ = 0.036	68 (20.3%)
Surgery duration (h)		8.4 ± 2.0	9.6 ± 2.2	0.002	*r* = 0.171	9.5 ± 2.2
Skin paddle						
	None	15 (40.5%)	134 (45.0%)	0.610	φ = 0.028	149 (44.5%)
	Intraoral	17 (45.9%)	108 (36.2%)	0.250	φ = 0.063	125 (37.3%)
	Extraoral	7 (18.9%)	61 (20.5%)	0.825	φ = 0.012	68 (20.3%)
Reconstructed regions						
	Condyle	5 (13.5%)	35 (11.7%)	0.755	φ = 0.017	40 (11.9%)
	Ramus	18 (48.6%)	93 (31.2%)	0.034	φ = 0.116	111 (33.1%)
	Body	36 (97.3%)	291 (97.7%)	1.000	φ = 0.007	327 (97.6%)
	Symphysis	23 (62.2%)	193 (64.8%)	0.755	φ = 0.017	216 (64.5%)

*φ = phi coefficient*,* r = pearson correlation coefficient*

The flap types used are shown in Table 3. The most common soft tissue flap used for bridging plate reconstructions was the ALT flap (78.4%), while the FFF was the predominant choice for osseous reconstructions (85.6%).

**Table 3:** Flap donor sites

Bridging Plate			Osseous Flap		
*n* = 37 (11.0%)			*n* = 298 (89.0%)		
ALT	LDF	RFF	FFF	SFF	DCIA
29 (78.4%)	5 (13.5%)	3 (8.1%)	255 (85.6%)	32 (10.7%)	11 (3.7%)

*FFF = fibula free flap*,* SFF = scapula free flap*,* DCIA = deep circumflex iliac artery*,* ALT = anterolateral thigh*,* LDF = latissimus dorsi*,* RFF = radial forearm flap*

Post-operative complication rates are shown in Table 4. Although all patients completed six-month follow-up, patients in the *bridging plate* group had a significantly shorter follow-up duration (20.9 ± 15.1 vs. 32.3 ± 20.3 months, *p* < 0.001). Furthermore, mortality during the follow-up period was significantly higher in this group (24.3% vs. 10.1%, *p* = 0.011), as was the incidence of pneumonia during the immediate postoperative ICU stay (13.5% vs. 4.7%, *p* = 0.029). Among maxillofacial complications, wound infections were significantly more frequent in patients who received osseous reconstructions (30.9% vs. 13.5%, *p* = 0.028). The length of hospital and ICU stays as well as all other observed complications, including flap loss and plate exposure, showed no significant differences between groups.

**Table 4:** Postoperative complication rates

	Bridging Plate	Osseous Flap	*p*-value	Effect Size	Overall
	*n* = 37 (11.0%)	*n* = 298 (89.0%)			*N* = 335
Follow-up (months)	20.9 ± 15.1	32.3 ± 20.3	< 0.001	*r* = 0.196	31.1 ± 20.1
Death	9 (24.3%)	30 (10.1%)	0.011	φ = 0.139	39 (11.6%)
ICU stay (days)	2.5 ± 5.0	2.4 ± 6.1	0.625	*r* = 0.070	2.4 ± 6.0
Hospital stay (days)	22.2 ± 13.5	20.6 ± 11.8	0.794	*r* = 0.014	20.7 ± 12.0
Pneumonia (on ICU)	5 (13.5%)	14 (4.7%)	0.029	φ = 0.119	19 (5.7%)
Early flap loss	2 (5.4%)	13 (4.4%)	0.773	φ = 0.016	15 (4.5%)
Late flap loss	0 (0.0%)	13 (4.4%)	0.196	φ = 0.071	13 (3.9%)
Plate exposure	8 (21.6%)	74 (24.8%)	0.669	φ = 0.023	82 (24.5%)
Fixation failure	2 (5.4%)	18 (6.0%)	0.878	φ = 0.008	20 (6.0%)
Wound infection (recipient site)	5 (13.5%)	92 (30.9%)	0.028	φ = 0.120	97 (29.0%)
Soft tissue complications	19 (51.4%)	159 (53.4%)	0.818	φ = 0.013	178 (53.1%)
Donor site complications	9 (24.3%)	97 (32.6%)	0.311	φ = 0.055	106 (31.6%)

*r = pearson correlation coefficient*,* φ = phi coefficient*,* ICU = intensive care unit*

**Fig. 2:**
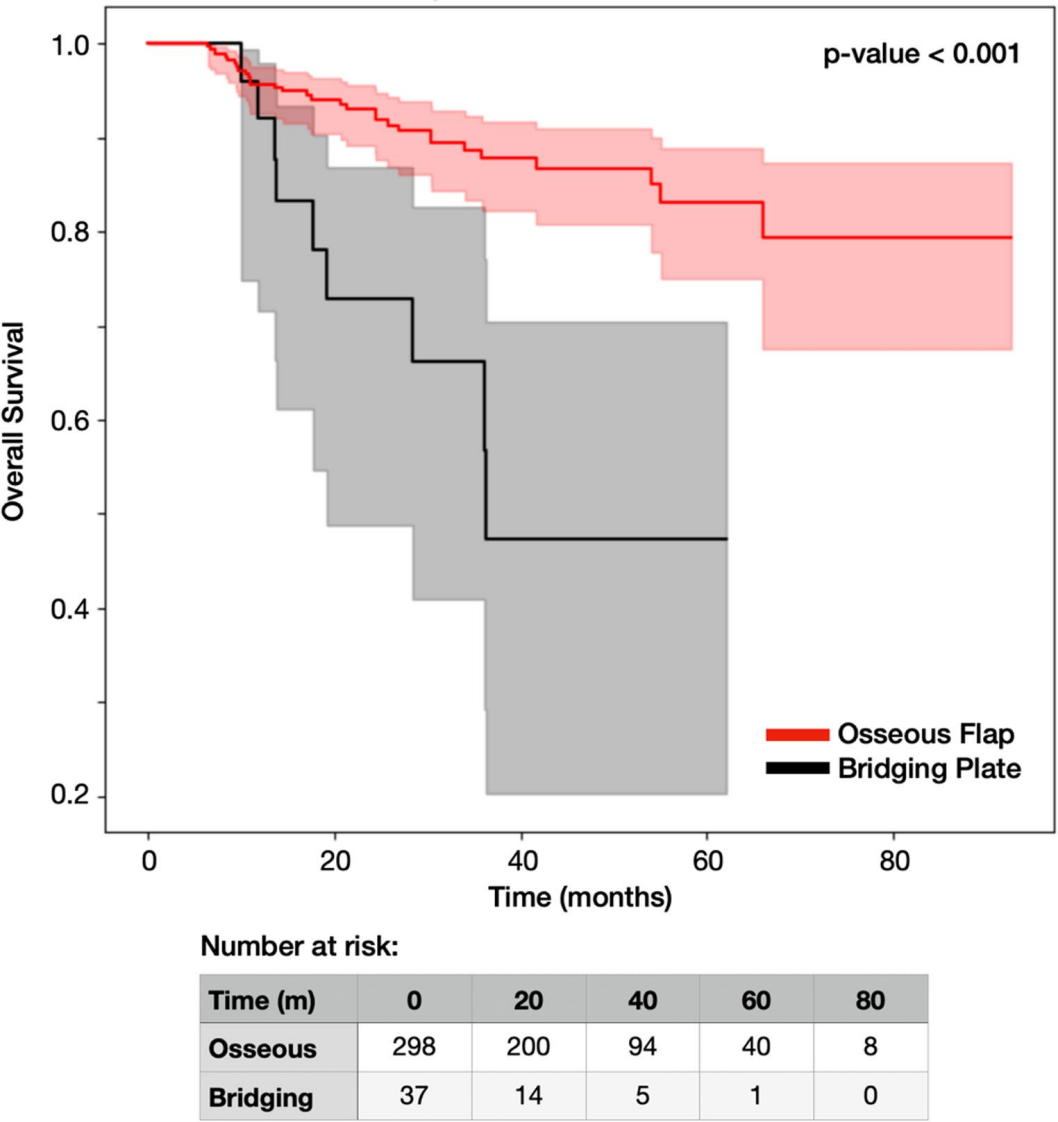
Kaplan-Meier survival curves and log-rank test comparing overall survival between osseous flap and bridging plate reconstruction groups. Shaded areas represent 95% confidence intervals

Kaplan-Meier analysis confirmed significantly lower overall survival in the bridging plate group (log-rank *p* < 0.001, Fig. 2).

A stratified analysis restricted to patients with malignant tumors (*n* = 256) showed consistent findings: mortality remained significantly higher in the bridging plate group (36.4% vs. 9.4%, *p* < 0.001), while plate exposure (27.3% vs. 23.9%, *p* = 0.727), fixation failure (0.0% vs. 6.0%, *p* = 0.239), and flap loss rates showed no significant differences (Supplementary Table 1).

## Discussion

This study compares postoperative complication rates after reconstruction of segmental mandibular defects using osseous flaps with bridging plate reconstructions. Our findings demonstrate that despite patients in the *bridging plate* group being significantly older and comorbid, both approaches achieve comparable rates of postoperative complications.

The higher frailty among bridging plate reconstructions most likely accounts for the significantly higher mortality (24.3% vs. 10.1%, *p* = 0.011) and postoperative pneumonia (13.5% vs. 4.7%, *p* = 0.029) rates. It is therefore important to emphasize that these adverse outcomes are not attributable to the surgical technique itself but rather to the patient condition that likely prevented the use of an osseous flap. The decision to perform a bridging plate reconstruction represents a pragmatic adaptation to patient-specific limitations, including salvage settings, allowing surgeons to restore mandibular continuity in patients who might otherwise be deemed unsuitable for any reconstructive procedure.

Our study challenges the prevailing assumption that bridging plate reconstructions inherently carry higher complication rates. The plate exposure rate in our cohort showed no significant difference between groups (21.6% vs. 24.8%, *p* = 0.669), contrasting with the meta-analysis by Bauer et al., which reported significantly higher exposure rates for bridging plate reconstructions [8]. This discrepancy may be attributed to our use of PS-3D reconstruction plates, which potentially offer superior soft tissue coverage through optimized plate design and positioning. The comparable fixation failure rates (5.4% vs. 6.0%, *p* = 0.878) further support the mechanical adequacy of modern PS-3D reconstruction plates in both scenarios.

The significantly shorter operative time for bridging plate reconstructions (8.4 ± 2.0 vs. 9.6 ± 2.2 h, *p* = 0.002) represents another advantage for this higher risk patient population. In a recent study among microvascular head and neck reconstructions, each additional hour of surgery duration increased postoperative complication rates by 13% (adjusted for confounders), with procedures exceeding nine hours resulting in twice as many complications compared to those under nine hours [15]. Additionally, the absence of functional donor site morbidity associated with bone harvest is a significant benefit, though our study showed no significant difference in overall donor site complications between groups [16–18].

Nevertheless, the observed difference in operating time in this study may have been influenced by differences in flap harvest technique. While FFF, RFF, and ALT flaps permit simultaneous two-team harvest without intraoperative repositioning, SFF and LDF flaps typically require intra-operative patient repositioning, extending operative time. In our cohort, the predominant use of ALT flaps (78.4%) in the bridging plate group and FFF flaps (85.6%) in the osseous group minimizes the confounding effect of patient repositioning on the observed time difference. The shorter operative time therefore primarily reflects the reduced complexity of soft tissue harvest compared to osseous flap harvest, which requires additional time for osteotomies, bone contouring, and precise positioning of the vascularized bone segments.

A notable finding was the higher rate of wound infections in the osseous reconstruction group (30.9% vs. 13.5%, *p* = 0.028). This is potentially related to the longer operative time, more extensive surgical dissection, and the significantly higher rate of adjuvant radiotherapy (41.3% vs. 24.3%, *p* = 0.047) in this group, which is a well-known risk factor for wound healing disturbances. However, this did not translate into increased flap loss rates, suggesting that infections were successfully managed with appropriate antibiotic therapy and local wound management.

The significantly shorter follow-up duration in the *bridging plate* group (20.9 ± 15.1 vs. 32.3 ± 20.3 months, *p* < 0.001) warrants consideration. This difference likely reflects the higher mortality rate in this elderly, multimorbid population. While this could theoretically result in underreporting of late complications, our minimum 6-month follow-up ensures capture of most acute postoperative events.

Our findings have important implications for clinical practice. First, they validate bridging plate reconstructions combined with soft tissue flaps as a viable option for selected patients, with outcomes that justify its use when osseous reconstruction is contraindicated or rejected by the patient. Second, they emphasize the importance of comprehensive preoperative assessment and individualized treatment planning. The presence of advanced age, cardiovascular comorbidities, or anticoagulation therapy should prompt consideration of less invasive reconstructive options.

Our results also lend new weight to a two-stage reconstructive approach. This study demonstrates that an initial bridging plate reconstruction can be performed with a shorter operative time and without an increased risk of plate-related complications like exposure or fixation failure. Given these findings, bridging plate reconstruction stands out as a robust initial procedure to safely and efficiently restore mandibular continuity, particularly in the frail patient population we identified. A secondary vascularized bone graft could then be planned for a later date, once the patient has recovered and is better able to tolerate a more extensive procedure. This staged strategy leverages the safety and speed of the initial bridging plate to address the immediate defect, while reserving the functionally superior osseous reconstruction for a time when the primary goal shifts to long-term dental rehabilitation.

Future directions should address the limitation of dental rehabilitation in non-osseous reconstructions. While recent innovations in plate design with integrated implant receptacles show promise, widespread clinical validation is needed [6, 7]. Additionally, the development of personalized risk stratification tools could help optimize patient selection and decision making.

This study has several strengths, including a large single-center cohort, standardized surgical protocols, and the exclusive use of patient-specific 3D-printed plates. However, the retrospective design and inherent selection bias in treatment allocation preclude causal inference. Multivariate regression analysis was not performed due to insufficient events in the bridging plate group (*n* = 37) for stable covariate adjustment, as logistic regression requires approximately 10 events per predictor variable. However, stratified analysis restricted to patients with malignant tumors demonstrated consistent findings, suggesting the observed associations are not solely driven by differences in treatment indication. The single-center nature may limit generalizability, though it ensures consistency in surgical technique and postoperative management. While our minimum 6-month follow-up captured acute complications, we acknowledge that the significantly shorter mean follow-up in the bridging plate group, likely driven by higher mortality in this frail population, limits the definitive assessment of long-term hardware-related complications.

## Conclusion

Our study demonstrates that bridging plate reconstructions of segmental mandibular defects combined with soft tissue flaps represent a valuable treatment option for carefully selected patients when osseous flaps are considered inappropriate due to age, comorbidities or disease progress. While these patients faced higher overall morbidity and mortality in our study due to their underlying conditions, postoperative complication rates did not increase with bridging plate reconstructions. Contrarily, osseous flaps had a significantly higher rate of wound infections, although this did not translate to increased rates of flap loss. Our findings support a personalized approach to mandibular reconstruction, where treatment selection is guided by individual patient factors.

## Supplementary Information

Below is the link to the electronic supplementary material.

Supplementary Material 1 (DOCX 18.8 KB)

## Data Availability

The datasets used and analysed during the current study are available from the corresponding author on reasonable request.

## Abbreviations

ALT: Anterolateral thigh flap
BMI: Body mass index
CV: Cramer’s V
DCIA: Deep circumflex iliac artery flap
FFF: Fibula free flap
ICU: Intensive care unit
LDF: Latissimus dorsi flap
MRONJ: Medication-related osteonecrosis of the jaw
ORN: Osteoradionecrosis
PS-3D: Patient-specific 3D-printed
RFF: Radial forearm flap
SFF: Scapula free flap
WHD: Wound healing disorder
