# Supplementary material for: Osseous free flap vs. Bridging plate mandibular reconstruction: a retrospective cohort study on perioperative complications of 335 patients
Source: Clin Oral Investig. 2026 Jan 17;30(1):56. doi: 10.1007/s00784-026-06753-7 (PMC12812072; doi:10.1007/s00784-026-06753-7)
Supplement: Supplementary file 1 — Supplementary Material 1 (DOCX 18.8 KB) [file 784_2026_6753_MOESM1_ESM.docx]

|  | **Bridging Plate** | **Osseous Flap** | **p-value** | **Effect Size** | **Overall** |
| --- | --- | --- | --- | --- | --- |
|  | **n = 22 (8.6%)** | **n = 234 (91.4%)** |  |  | **N = 256** |
| **Follow-up (months)** | 18.9 ± 12.8 | 32.7 ± 20.6 | < 0.001 | r = 0.208 | 31.5 ± 20.4 |
| **Death** | 8 (36.4%) | 22 (9.4%) | < 0.001 | φ = 0.235 | 30 (11.7%) |
| **ICU stay (days)** | 2.8 ± 6.4 | 2.5 ± 6.7 | 0.962 | r = 0.003 | 2.5 ± 6.7 |
| **Hospital stay (days)** | 25.2 ± 15.4 | 20.7 ± 12.4 | 0.207 | r = 0.079 | 21.1 ± 12.7 |
| **Pneumonia (on ICU)** | 3 (13.6%) | 12 (5.1%) | 0.105 | φ = 0.012 | 15 (5.9%) |
| **Early flap loss** | 1 (4.5%) | 8 (3.4%) | 0.561 | φ = 0.017 | 9 (3.5%) |
| **Late flap loss** | 0 (0.0%) | 13 (5.6%) | 0.257 | φ = 0.071 | 13 (5.1%) |
| **Plate exposure** | 6 (27.3%) | 56 (23.9%) | 0.727 | φ = 0.022 | 62 (24.2%) |
| **Fixation failure** | 0 (0.0%) | 14 (6.0%) | 0.239 | φ = 0.074 | 14 (5.5%) |
| **Wound infection (recipient site)** | 3 (13.6%) | 71 (30.3%) | 0.099 | φ = 0.103 | 74 (28.9%) |
| **Soft tissue complications** | 11 (50.0%) | 122 (52.1%) | 0.848 | φ = 0.012 | 133 (52.0%) |
| **Donor site complications** | 4 (18.2%) | 79 (33.8%) | 0.136 | φ = 0.093 | 83 (32.4%) |

**Supplementary Table 1** Postoperative complication rates among patients operated for malignant tumors only
